# Direct Anterior Approach in Total Hip Arthroplasty Leads to Superior Outcomes at 3-Month Follow-up When Compared With the Posterior Approach: A Matched Study Using Propensity Score Analysis

**DOI:** 10.5435/JAAOSGlobal-D-19-00118

**Published:** 2019-12-23

**Authors:** David R. Maldonado, Joseph R. Laseter, Cynthia Kyin, Ajay C. Lall, Benjamin G. Domb

**Affiliations:** From the American Hip Institute (Dr. Maldonado, Ms. Kyin, Dr. Lall, Dr. Domb), Des Plaines, IL, and the Case Western Reserve University School of Medicine (Mr. Laseter), Cleveland, OH.

## Abstract

The purpose of this study was to report and compare early outcomes during the first 3 months of the recovery phase in patients who underwent primary total hip arthroplasty (THA) with direct anterior approach (DAA) and posterior approach (PA).

**Background:**

The DAA for primary THA has gained popularity within the past few years. Although controversy exists regarding the long-term benefit when compared with the PA, several authors have reported markedly better outcomes in the early recovery weeks, when using DAA.

**Methods:**

For this study, data were prospectively collected and retrospectively reviewed for all primary THAs from March 2014 to October 2017. Included patients underwent primary THA through DAA or PA and had minimum 3-month postoperative measures for the Harris Hip Score, Forgotten Joint Score-12, Veterans RAND 12 Mental (VR-12 Mental), Veterans RAND 12 Physical (VR-12 Physical), 12-Item Short-Form (SF) Survey Mental, 12-Item SF Survey Physical (SF-12 Physical), Visual Analog Scale, and patient satisfaction. An analysis using propensity score matching was done to establish the DAA and PA groups. Matching (1:1 ratio) was conducted based on the following covariates: age, sex, body mass index, and laterality.

**Results:**

Twenty-four DAA THA patients were successfully matched using propensity scoring to 24 PA THA patients. The DAA group demonstrated significantly higher scores for the following patient-reported outcome scores: Harris Hip Score, VR-12 Mental, VR-12 Physical, and SF-12 Physical (*P* = 0.0090, *P* = 0.0388, *P* = 0.0063, and *P* = 0.0132, respectively).

**Conclusion:**

At 3-month follow-up, both the DAA and PA groups reported favorable outcomes after THA. However, the DAA group scored markedly higher regarding quality-of-life outcomes when compared with a propensity score–matched group of PA patients.

Total hip arthroplasty (THA) is one of the most successful orthopaedic procedures.^1(p2),2,3^ However, controversy regarding the ideal approach for THA remains.^4^ Recent literature demonstrating that the direct anterior approach (DAA) for primary THA may offer advantages in the early postoperative period has spurred an increase in DAA popularity.^1,5,6^ These studies have shown less pain, lower blood loss, and shorter hospital stay with the DAA when compared with other approaches such as the posterior approach (PA).^5,7,8,9,10^ The purpose of this study was to report and compare early outcomes during the first 3 months of the recovery phase in patients who underwent primary THA with DAA and PA. We hypothesized that patients who underwent primary THA with DAA would experience markedly higher outcomes during the first 3 months after surgery than patients who underwent primary THA with PA.

## Methods

### Participation in the Blinded Hip Preservation Registry

Although this study represents a unique analysis, data on some patients in this study may have been reported in other studies.

### Patient Selection Criteria

The data were prospectively collected and retrospectively reviewed for all patients who underwent primary THA by the senior author (B.G.D.). The date range for this study was March 2014 to October 2017.

Patients chosen for this study were included if they underwent a DAA or PA THA and had 3-month patient-reported outcome scores (PROs). Full follow-up included scores for the following outcome measures: Harris Hip Score (HHS), Forgotten Joint Score-12 (FJS-12), Veterans RAND 12 Mental (VR-12 Mental), VR 12 Physical (VR-12 Physical), 12-Item Short-Form Survey Mental (SF-12 Mental), 12-Item SF Survey Physical (SF-12 Physical), Visual Analog Scale (VAS), and patient satisfaction. Any revision surgeries or complications were also documented. This study was approved by an institutional review board.

### Indications for Surgery

All patients received a diagnosis of hip osteoarthritis based on their medical history, physical examination, and imaging findings. If patients still presented with a concerning level of pain after documented conservative treatment, they were then scheduled for hip surgery by the senior surgeon (B.G.D.). To avoid unnecessary surgical intervention, all patients were required to undergo 3 months of conservative treatment options such as physical therapy sessions, intra-articular ultrasound-guided cortisone injections, rest, and anti-inflammatory drugs. In addition, after scheduling, patients in both groups underwent the same preoperative education program to further discuss the procedure and the postoperative protocol.

### Surgical Techniques

As previously described, all included patients received a THA through either a DAA or PA.^6^ All THAs were preformed with the patient under the same general anesthesia protocol regardless of group. Regarding the DAA, a traction table was used, and patients were placed in the supine position. A “T”-shaped incision was used to open the capsule, and absorbable sutures were used to close. Fluoroscopic guidance was used to assist with acetabular reaming and cup component placement.^11^ The implants used were the same for both groups.

For the PA, all THAs were conducted with the patient in the lateral position. The external rotators were identified and taken down for exposure while preserving the piriformis tendon whenever possible. The capsule was identified and incised in an inverted “L”-shaped fashion as a separate layer from the external rotators. At closing, the capsule was repaired using a transosseous repair with nonabsorbable sutures.^12,13^ In addition, a transosseous repair with nonabsorbable sutures was used to restore the external rotators.

### Rehabilitation

Patients in both groups were given the same perioperative protocols. For the weeks after their operation, patients in both groups were also given the same instructions and were directed to participate in at home care for 1 to 2 weeks with the use of a walker. Hip precautions were not given to patients in either group. This was followed by outpatient physical therapy for an additional 6 to 8 weeks to improve range of motion and strength. Patients would then also return for follow-up appointments with radiographic evaluation at the 2-week, 3-month, and annual time points.

### Surgical Outcomes

Postoperative PROs were obtained 3 months after the patients' procedures. The collected measures that were rated on a scale from zero to 100 were the HHS, FJS-12, VR-12 Mental, VR-12 Physical, SF-12 Mental, and SF-12 Physical. In addition, the VAS was used to evaluate pain level on a scale from zero (no pain) to 10 (worst possible pain), and patient satisfaction was rated of 10 (10 = highest satisfaction). Outcome results were obtained through the following methods: clinical appointment, telephone interview, or encrypted e-mail.

### Statistical Analysis

Other than propensity score matching, all analyses were completed using Microsoft Excel (Microsoft) with the Real Statistics resource pack. Normality and variance were assessed for all continuous variables using the Shapiro-Wilk test and *F*-test, respectively. Analysis of the data were then conducted through a Student *t*-test or a nonparametric equivalent. Calculated mean values and SDs are also reported for all continuous data. Regarding categorical data, all measurements were compared using a Fisher exact test. A *P* value <0.05 was considered statistically significant.

Propensity score matching was done using R (Version 3.4.0; R Foundation for Statistical Computing). This method of matching was used to limit the effect of potential confounding variables when comparing the DAA with PA.^14,15^ The algorithm used established matches based on the logit of the propensity score with a 0.20 SD caliper width.^16^ The style of matching used to match DAA patients to PA patients was greedy matching without replacement in ;a one-to-one ratio. Therefore, if a DAA patient was matched to a PA patient, this DAA patient could not be reassigned to a different PA patient. This method of matching has been established in the literature as an optimal method for estimating differences between treatment groups.^14^

The variables that the DAA and PA groups were matched on were age, sex, body mass index (BMI), and laterality. A logistic regression was used to estimate the propensity scores, and each score represented the probability of that patient receiving a DAA or PA hip arthroplasty. Imbalances between the DAA and PA groups were detected by comparing standardized mean differences (SMDs) before and after matching. If their respective SMD was >0.01, a group was considered imbalanced for that covariate.^17^ *P* values were also calculated for each of the covariates to determine whether there were significant differences between the groups both before and after matching.

## Results

### Patient Demographics

**Figure 1:**
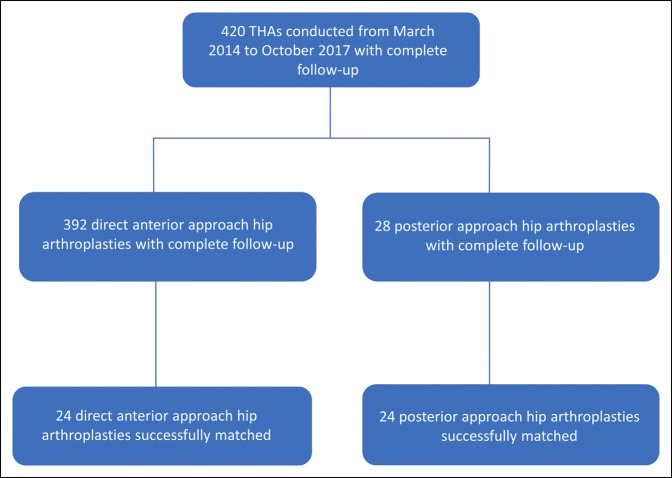
Flowchart of patient selection. THA = total hip arthroplasty.

From March 2014 to October 2017, there were a total of 420 THAs with 3-month follow-up. Of these surgeries, 392 hips in 369 patients had a DAA THA and 28 hips in 28 patients had PA THA, Figure 1.

**Figure 2:**
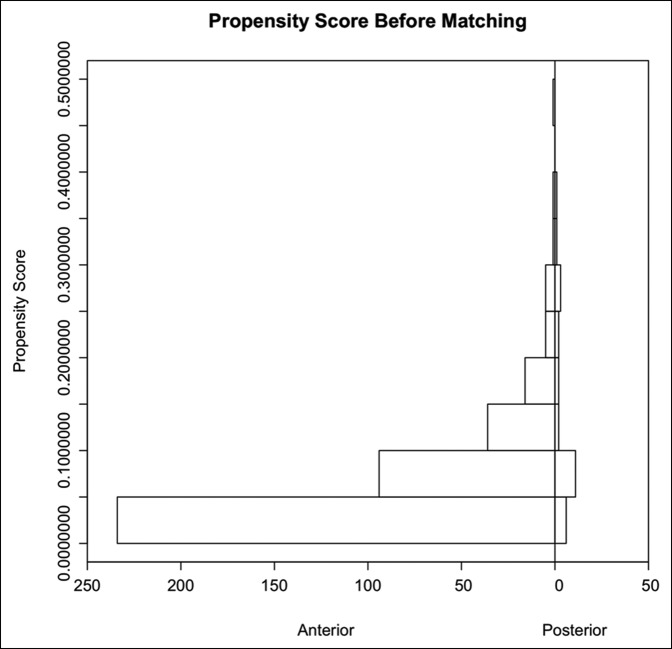
Back-to-back histogram illustrating the distributional similarity of the 2 groups before matching.

**Figure 3:**
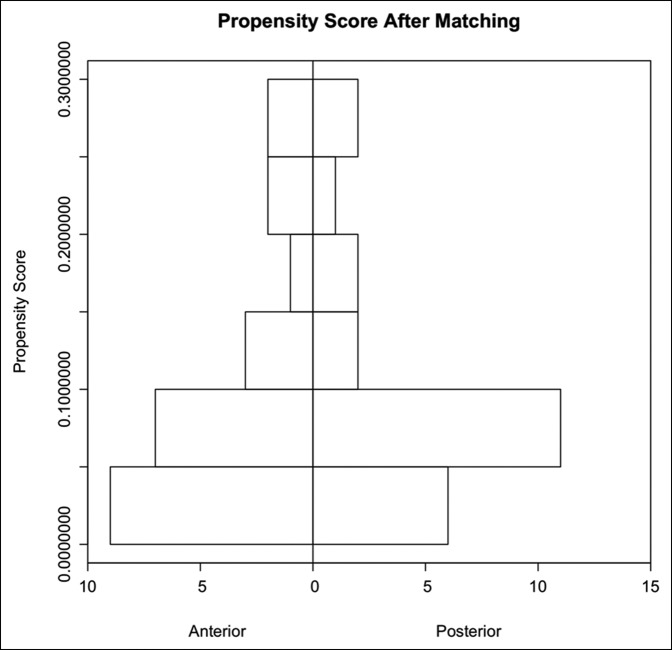
Back-to-back histogram illustrating the improvement in distributional similarity of the 2 groups after matching.

When matched for age, sex, BMI, and laterality, 24 patients were successfully matched using propensity scoring. Before matching, the DAA and PA groups were imbalanced regarding age, sex, BMI, and laterality with SMD values of 2.0515, 0.2335, 3.1897, and 0.1188, respectively. However, only sex and BMI were statistically different (*P* = 0.0162 and *P* = 0.0122, respectively). After matching, a residual imbalance remained regarding age and BMI (SMD = 1.2017 and SMD = 0.2150, respectively), but there were no longer significant differences regarding sex and BMI (*P* = 1.0 and *P* = 0.9005, respectively), Table 1. In addition, Figures 2 and 3 represent how the groups became more alike after matching. More specifically, the improvement in symmetry shows how the groups became more similar in terms of propensity score, thus demonstrating a decrease in selection bias.^18^

**Table 1:** Groups Demographics Matched

Factor	Anterior (n = 24)	Posterior (n = 24)	*P* value	SMD
Patients and hips included in study			1.0	0.000
Left	9 (37.5%)	9 (37.5%)		
Right	15 (62.5%)	15 (62.5%)		
Sex			1.0	0.000
Male	6 (25.0%)	6 (25.0%)		
Female	18 (75.0%)	18 (75.0%)		
Age at surgery (y, mean, SD, range)	58.9 ± 11.1 (37.7–79.1)	60.1 ± 9.3 (42.7–74.7)	0.6851	1.2017
BMI (kg/m^2^, mean, SD, range)	30.9 ± 6.2 (21.4–44.3)	31.2 ± 5.6 (19.8–39.2)	0.9005	0.2150
Follow-up time (mo, mean, SD)	3.2 ± 1.1	3.4 ± 1.9	0.8528	

BMI = body mass index, n = sample size, SMD = standardized mean difference

Although patients were not matched based on the setting in which they underwent THA, there were no notable differences between the groups in terms of the proportion of patients who underwent THA in the inpatient or outpatient setting. The number of patients undergoing an inpatient THA was 19 and 14 for the DAA and PA groups, respectively (*P* = 0.1195).

### Clinical Outcomes

At 3-month follow-up, the DAA group demonstrated significantly higher scores for the following PROs: HHS, VR-12 Mental, VR-12 Physical, and SF-12 Physical (*P* = 0.0090, *P* = 0.0388, *P* = 0.0063, and *P* = 0.0132, respectively), Table 2. However, no significant differences were found regarding FJS-12 and SF-12 Mental (*P* = 0.0624 and *P* = 0.2353, respectively).

**Table 2:** Matched Patient-Reported Outcome Scores

Factor	Anterior	Posterior	*P* value
HHS	79.8 ± 16.6	65.7 ± 19.1	0.0090
FJS-12	59.5 ± 32.8	41.1 ± 33.9	0.0624
VR-12 Mental	60.1 ± 6.7	54.7 ± 10.5	0.0388
VR-12 Physical	45.9 ± 11.5	36.5 ± 11.3	0.0063
SF-12 Mental	56.5 ± 8.0	53.3 ± 11.0	0.3436
SF-12 Physical	42.9 ± 11.5	34.7 ± 10.9	0.0132

HHS = Harris Hip Score, FJS-12 = Forgotten Joint Score-12

VR-12 Physical and VR-12 Mental, Veterans RAND 12-Item Health Survey both physical and mental; SF-12 Physical and SF-12 Mental, Health Survey Short Forms both physical and mental.

**Figure 4:**
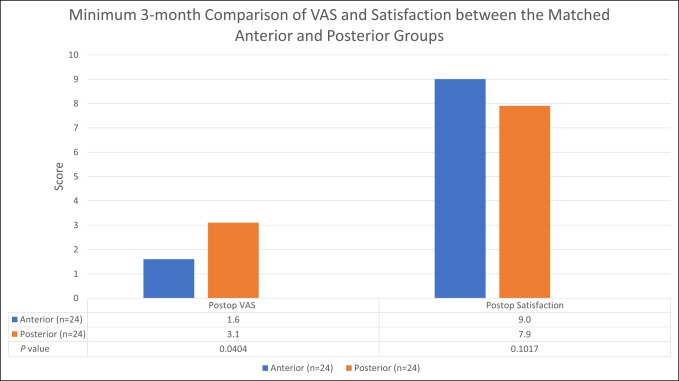
Bar graph comparing the 3-month postoperative Visual Analog Scale (VAS) and patient satisfaction scores between the anterior and posterior groups. Postop = postoperative, n = sample size

Regarding VAS and patient satisfaction, the DAA group demonstrated significantly better scores for VAS (*P* = 0.0478) but not satisfaction (*P* = 0.1680). Comparatively, the DAA group had an average VAS score of 1.7 ± 2.1 versus 3.1 ± 2.7 for the PA group. Complete data regarding VAS and patient satisfaction are reported in Figure 4.

### Complications and Revisions

Overall, 1 (4.2%) complication occurred in the DAA group. This patient had a sciatic nerve injury which resolved over time. Regarding the PA group, there were zero complications. In addition, none of the included patients required a revision surgery.

## Discussion

This study demonstrated that at 3-month follow-up, patients who underwent a primary DAA THA achieved markedly higher scores in the early postoperative period for the HHS, VR-12 Mental, VR-12 Physical, SF-12 Physical, and VAS than a matched PA THA group.

In a recent single-blinded randomized study, Christensen and Jacobs^5^ compared 28 primary DAA THAs with 23 primary PA THAs and found that the DAA cohort had significantly better results concerning pain scores at the 6-week follow-up timepoint (*P* < 0.05). This study demonstrated similar results where the DAA group showed significantly better results regarding VAS (*P* = 0.0404). This finding may be due to the lesser muscle damage associated with using natural intermuscular intervals.^19–21^

Parvizi et al. has also shown early advantages of the DAA over other popular approaches. Recently, they conducted a randomized study comparing the DAA with the direct lateral approach and found that the DAA group had markedly earlier functional independence. These results could be because the DAA approach does not require the detachment of any musculature, thus preserving the abductor mechanism arm.^8^ In addition, a randomized study by Taunton et al. compared the DAA and PA in high-volume THA surgeons and also reported better outcomes for the DAA group. They found that the DAA group required the use of a walker for less time (10 versus 15 days, *P* = 0.01) and discontinued all walking aids at a faster rate than the PA group (17 versus 24 days, *P* = 0.04).^9^

Regarding PROs, our study showed a higher average HHS value (*P* < 0.05) for the DAA group over the PA at a minimum 12-week follow-up. Similar results were also published by Graves et al. who compared 86 primary DAA THAs with 145 primary PA THAs. Using the VR-12 Physical outcome score, they concluded that the DAA group had a higher improvement in physical function (*P* = 0.008).^22^

The strengths of the current study must be acknowledged. First is our use of multiple validated functional hip outcome scores after THA. Second, this is one of the few studies comparing early PROs of DAA and PA in primary THA using a propensity score matching strategy. Third, all surgical procedures were conducted by a single, high-volume, fellowship-trained surgeon, which makes the learning curve, especially with DAA, a nonissue and thus not a confounding variable.^23,24^

However, this study has several limitations, and the results should be interpreted with caution. First, this is a nonrandomized study and includes a retrospective design. Second, although propensity matching was used, other confounding variables such as comorbidities were not matched on and may have influenced our results. Third, the sample size is small regarding the PA group, and the study may be underpowered. Fourth, although this study provides PROs for the 3-month period, data from earlier timepoints such as the first postoperative or 6-week visits were not recorded. Finally, this study includes surgical procedures done by a single, high-volume, fellowship-trained surgeon, and the learning curve associated with DAA for THA may have made the overall outcomes not generalizable.^6,23–25^

## Conclusion

At 3-months follow-up, both the DAA and PA groups reported favorable outcomes after THA. However, the DAA group scored markedly higher regarding quality-of-life outcomes when compared with a propensity score–matched group of PA patients.
